# Totally extraperitoneal approach to laparoscopic lateral lymph node dissection for patients with recurrent lateral pelvic lymph nodes after rectal cancer surgery: a novel technique—M TEP LLND

**DOI:** 10.1007/s00595-019-01808-7

**Published:** 2019-04-10

**Authors:** Shinsuke Masubuchi, Junji Okuda, Hiroki Hamamoto, Masatsugu Ishii, Wataru Osumi, Masashi Yamamoto, Yoshihiro Inoue, Keitaro Tanaka, Kazuhisa Uchiyama

**Affiliations:** 1 Department of General and Gastroenterological Surgery, Osaka Medical College, 2-7 Daigaku-machi, Takatsuki, 569-8686 Japan; 2 Cancer Center, Osaka Medical College, Takatsuki, 569-8686 Japan

**Keywords:** Extraperitoneal approach, Lateral lymph node dissection, Totally extraperitoneal (TEP)

## Abstract

Lateral lymph node dissection (LLND) for recurrence of lateral pelvic lymph node metastasis after rectal cancer surgery is technically demanding because of the need for re-do surgery. We herein report a novel technique of laparoscopic LLND via a totally extraperitoneal (TEP) approach. Since October 2018, we have performed LLND based on a TEP approach, called “M TEP LLND”, with two cases treated. By peeling in the caudal direction in the dorsal layer of the rectus abdominis muscle, a working space is created once the extraperitoneal space is reached, and LLND is performed. All lateral pelvic lymph node dissection procedures have been successfully completed, and there have been no intraoperative or postoperative complications. This procedure allows TEP-experienced colorectal surgeons to perform safe and complete LLND without any influence of intraperitoneal adhesion or intestinal obstruction. M TEP LLND is less invasive than the conventional intraperitoneal approach and appears to be useful, particularly for recurrence of lateral pelvic lymph node metastasis.

## Introduction

Patients with local recurrence after rectal cancer surgery have a poor prognosis. Lateral pelvic lymph node metastasis is one of the major causes of local recurrence after total mesorectal excision (TME) [1, 2]. Local recurrence limited to lateral pelvic lymph nodes can be potentially treated by salvage lateral pelvic lymph node dissection (LLND). The safety and feasibility of laparoscopic LLND with TME for primary rectal cancer have been reported, but only a few studies have assessed laparoscopic salvage LLND for recurrent lateral pelvic lymph nodes [3–6]. LLND for recurrence of lateral pelvic lymph node metastases is technically demanding because of the need for re-do surgery.

We herein report a novel technique via a totally extraperitoneal (TEP) approach to laparoscopic LLND for patients with recurrent lateral pelvic lymph nodes after rectal cancer surgery.

## Material and methods

Since October 2018, we have been performing LLND with a TEP approach, called “M TEP LLND”, for patients with resectable lateral lymph node recurrence after rectal cancer surgery; two cases have been experienced thus far.

**Fig. 1:**
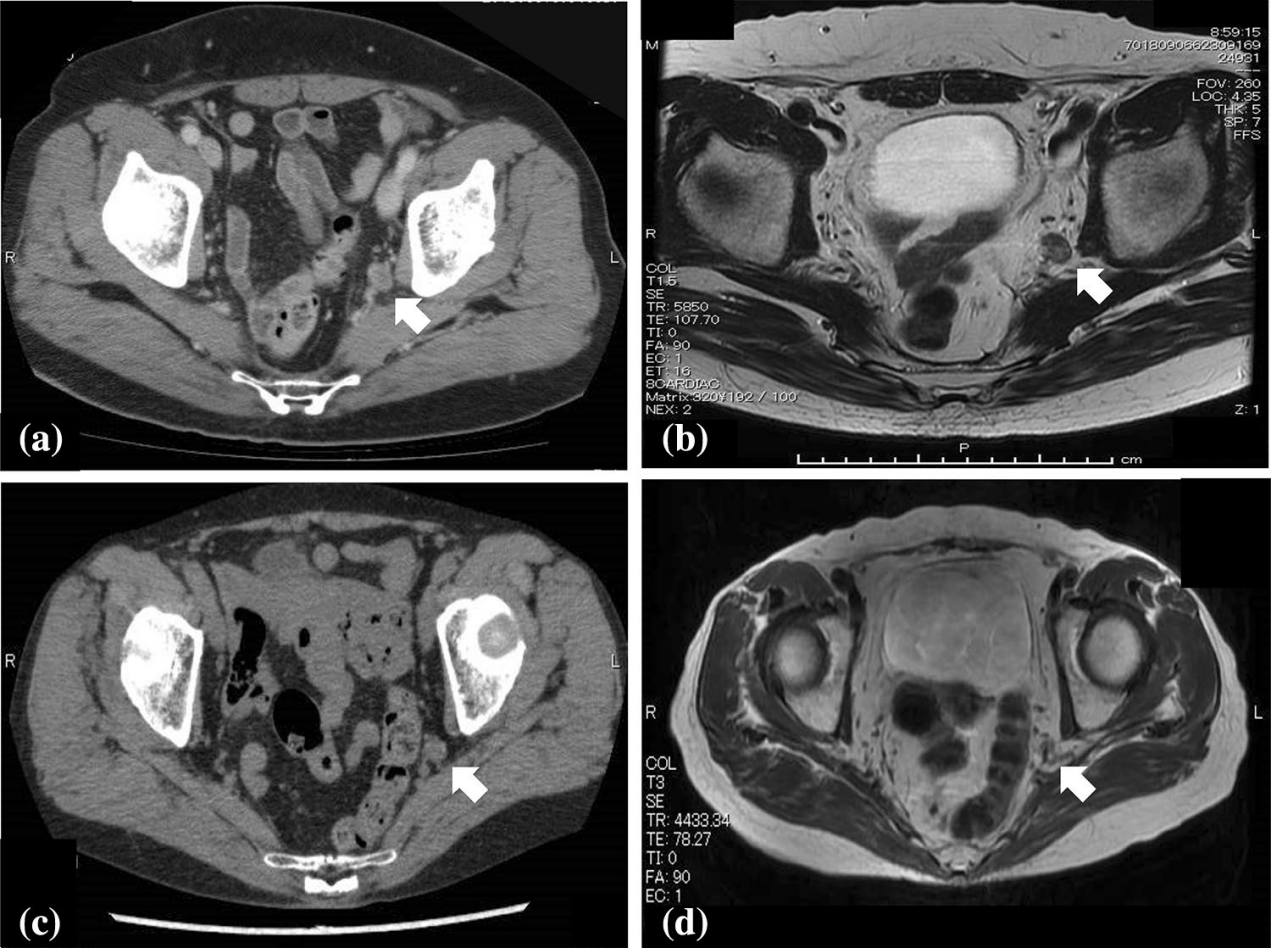
Representative abdominal computed tomography (CT) and magnetic resonance imaging (MRI) findings of patients 1 and 2. **a** Follow-up CT of patient 1 after primary surgery revealed left lateral pelvic lymph node recurrence. **b** Follow-up MRI of patient 1 after primary surgery revealed left lateral pelvic lymph node recurrence. **c** Follow-up CT of patient 2 after primary surgery revealed left lateral pelvic lymph node recurrence. **d** Follow-up MRI of patient 2 after primary surgery revealed left lateral pelvic lymph node recurrence

Patients with local recurrence limited to lateral pelvic lymph nodes with the likelihood of an R0 resection are candidates for M TEP LLND. All patients were evaluated by abdominal computed tomography (CT) regularly during follow-up after the initial surgery. Patients who were found to have enlarged lateral pelvic lymph nodes underwent magnetic resonance imaging (MRI), and treatment was discussed at our board conference. Patient 1 was a 69-year-old man who had undergone laparoscopic intersphincteric resection without LLND 7 months earlier. We diagnosed left lateral pelvic lymph node recurrence pathologically as one positive node. Patient 2 was a 73-year-old woman who had undergone laparoscopic low anterior resection without LLND 1 year and 8 months earlier. We diagnosed left lateral pelvic lymph node recurrence pathologically as one positive node (Table 1). The CT and MRI images are shown in Fig. 1.

**Table 1:** Characteristics of patients

	Patient 1	Patient 2
Sex	Male	Female
Age (years)	69	73
BMI	31.6	21.5
Primary operation	Laparoscopic intersphincteric resection	Laparoscopic low anterior resection
Histology	Moderately differentiated adenocarcinoma	Well-differentiated adenocarcinoma
Pathological stage of primary cancer	Stage I	Stage II
Interval between primary surgery and salvage surgery (months)	7	20

*BMI* body mass index

### Surgical technique

**Fig. 2:**
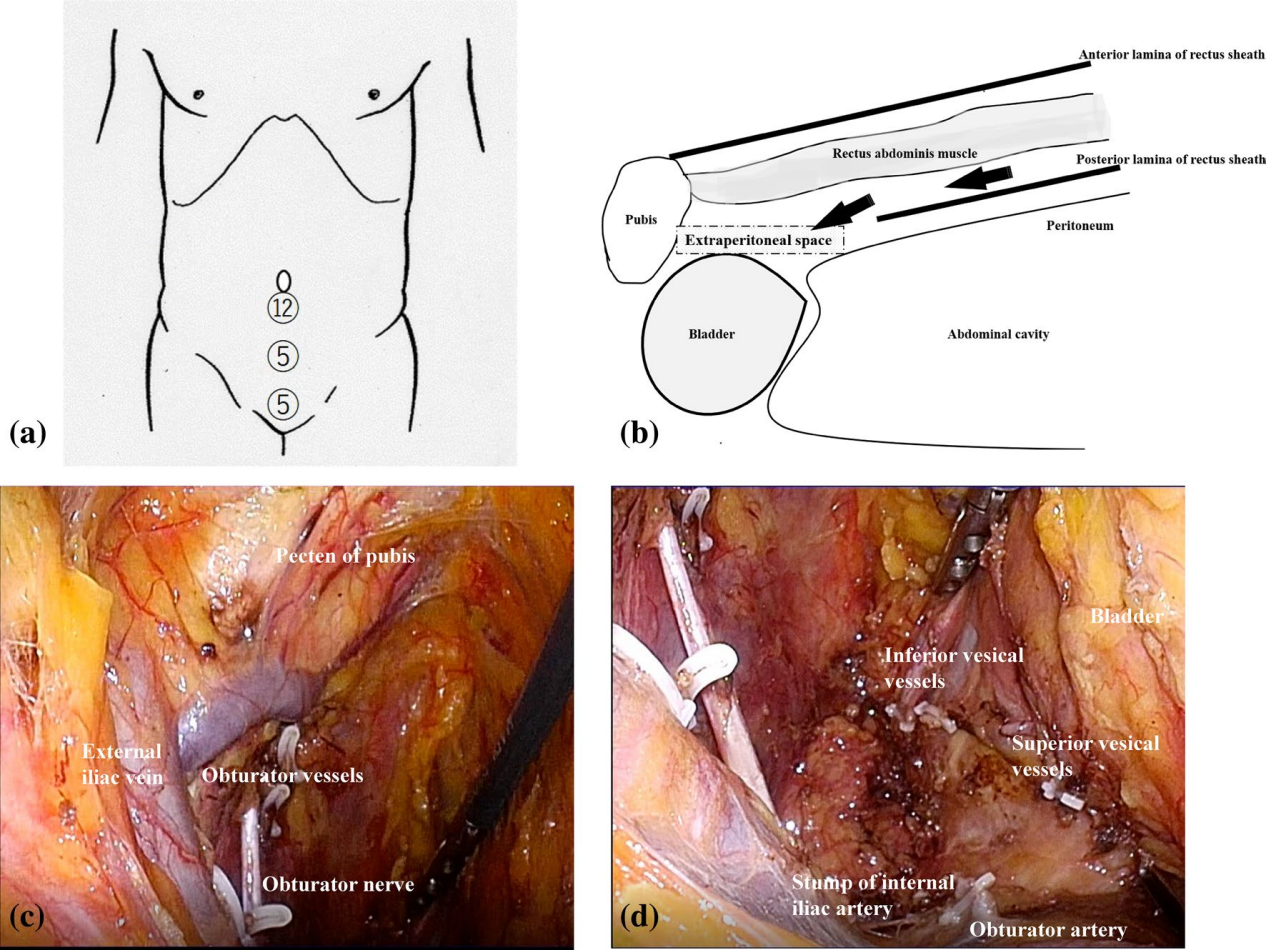
**a** Port placement.** b** Approach to the extraperitoneal space. We peeled toward the caudal direction in the dorsal layer of the rectus abdominis muscle and reached the extraperitoneal space.** c**, **d** Laparoscopic view after lateral pelvic lymph node dissection

This procedure is based on the TEP method for inguinal hernia surgery [7]. It begins with a small incision (about 15 mm) below the umbilicus. The incision of the anterior sheath of the rectus abdominis muscle is then made. Next, the rectus abdominis muscle is laid from the midline to the outside, and a 12-mm port is inserted in front of the posterior lamina of the rectus sheath. A camera is inserted through this port, and a 5-mm port is inserted in the midline between the umbilicus and the pubis. Peeling toward the caudal direction in the dorsal layer of the rectus abdominis muscle is performed, and on reaching the extraperitoneal space, and a 5-mm port is inserted 1 cm superior the median pubis (Fig. 2a, b).

First, with the pubic bone and Cooper’s ligament as landmarks, the external iliac vein is exposed. On identifying the obturator foramen, the obturator nerve is preserved, but the obturator vessels are divided. The extent of lymph node dissection is along the obturator internus muscle at the outer edge and along the bladder at the inner edge. The distal side of the dissection line is at Alcock’s canal. The branches of the internal iliac artery are clipped and divided (ex. superior vesical artery, inferior vesical artery, obturator artery, artery to the ductus deferens or uterine artery). The surface of the internal iliac vein is exposed, and the branches of the internal iliac vein are also clipped and divided. In cases in which the metastatic lateral pelvic lymph nodes have adhered or are close to the internal iliac artery, the artery is resected en bloc along with the lateral pelvic lymph nodes. The dissected lymph node is placed in the bag and removed from the 12-mm port wound (Fig. 2c, d).

## Results

The perioperative data are summarized in Table 2. The operation time was 231 min for patient 1 and 243 min for patient 2. The estimated blood loss was 10 ml in both. The adjacent structures removed en bloc were only the internal iliac artery in patient 1. R0 resection was achieved in both patients. The number of retrieved lymph nodes was seven for patient 1 and nine for patient 2. In both, the number of metastatic lymph nodes was 1. The postoperative length of hospital stay was 8 days for patient 1 and 10 days for patient 2. There were no postoperative complications.

**Table 2:** Perioperative data

	Patient 1	Patient 2
Operative time (min)	231	243
Blood loss (ml)	10	10
Adjacent structures removed en bloc	Internal iliac artery	None
Number of retrieved lymph nodes	7	9
Number of positive lymph nodes	1	1
Postoperative complication	None	None
Postoperative hospital stay (days)	8	10

## Discussion

Laparoscopic surgery provides a highly detailed image and magnifying effect, so it is extremely useful in cases of complicated surgery, such as salvage LLND performed in a very deep, narrow pelvic space. To our knowledge, few studies have focused on salvage surgery for recurrence of lateral pelvic lymph node metastasis after rectal cancer surgery [6].

Laparoscopic salvage LLND has been reported as a minimally invasive surgery because of its low blood loss [6, 8]. However, the influence of re-operation must also be considered, as adhesion of the small intestine and reconstructed colon to the pelvis makes laparoscopic LLND more difficult. However, the extraperitoneal approach allows TEP-experienced colorectal surgeons to perform safe and complete LLND without any adverse effects of intraperitoneal adhesion and intestinal obstruction.

In the present cases, we were able to remove all of the lymph nodes around the internal iliac artery, external iliac artery, and obturator nerve. However, this technique has limited use when applied to dissection of lymph nodes around the common iliac artery. This technique can be performed as solo surgery without an assistant port. However, in our cases, surgery was performed with adequate staffing and the ability to transition to the intraperitoneal approach at any time to cope with unexpected intraoperative bleeding. There have been only a few reports of laparoscopic pelvic lymph node dissection via an extraperitoneal approach, so a thorough anatomical understanding is necessary for this approach [9, 10].

The technique reported this time is based on the TEP hernia repair method for inguinal hernia surgery, and we have termed it “M TEP LLND”. M TEP LLND is less invasive than the conventional intraperitoneal approach and appears to be useful, particularly for recurrent lateral pelvic lymph node metastasis. In this report of this method, the number of cases was small, and the observation period was short. In the future, it will be necessary to assess the long-term oncological outcomes of this method.

## Conclusion

The TEP approach to laparoscopic lateral lymph node dissection for patients with recurrent lateral pelvic lymph nodes after rectal cancer surgery is technically feasible and safe when performed by TEP-experienced colorectal surgeons. Although the short-term outcomes in the present cases were acceptable, the study was limited by its small size, and further investigations are needed to assess the oncological long-term outcomes of this technique.
